# Dispensaries for Hop-Pickers

**Published:** 1920-09-18

**Authors:** C. F. Tonks

**Affiliations:** Secretary, C.E.T.S. 64 Burgate, Canterbury.


*To the Editor of* The Hospital.

### Dispensaries for Hop-pickers.

*To the Editor of* The Hospital.

Sir,—The mission work among the hop-pickers was necessarily and rightly curtailed from 1914-18, and last year it was not normal, but this season the hop-picking will be on a pre-war scale, and the mission work is being arranged accordingly. Nurses are being provided and dispensaries opened; Sunday schools, lantern and other services are being arranged; concerts and entertainments will be provided; coffee-stalls and barrows are being organised to supply much-needed and wholesome refreshments.

A large body of missioners, men and women, will devote the next few weeks to work among the pickers, and these will include a body of students from King's College, London, which is this year initiating a College Mission, under the direction of the C.E.T.S. Hop-pickers' Mission, at an important picking centre near Faversham. This Mission will cost at least £200 this year, and we specially appeal for funds. Probably old students of King's College would like specially to assist in providing the charges of the Mission district being worked by King's College, London, men. Gifts of linen for bandages, toys for the younger children, and readable literature will be gratefully received at the Offices of the Mission: 64 Burgate Street, Canterbury.

No one who has ever spent a season among the hop-pickers will fail to appreciate the part the Mission plays in making these two or three weeks in Kent profitable, health-giving, and beneficial to the dwellers in East London and other populous areas.—I am, Sir,
Yours faithfully,
C. F. Tonks,
Secretary, C.E.T.S.
64 Burgate, Canterbury.

